# Outcome of Extracorporeal Shock Wave Therapy for Insertional Achilles Tendinopathy with and without Haglund's Deformity

**DOI:** 10.1155/2016/6315846

**Published:** 2016-11-30

**Authors:** Ziying Wu, Wei Yao, Shiyi Chen, Yunxia Li

**Affiliations:** Department of Sports Medicine, Huashan Hospital, Fudan University, Shanghai, China

## Abstract

*Purpose.* To compare the results of extracorporeal shock wave therapy (ESWT) for insertional Achilles tendinopathy (IAT) with or without Haglund's deformity.* Methods.* Between September 2014 and May 2015, all patients who underwent ESWT were retrospectively enrolled in this study. A total of 67 patients were available for follow-up and assigned into nondeformtiy group (*n* = 37) and deformtiy group (*n* = 30). Clinical outcomes were evaluated by VISA-A Score and 6-point Likert scale.* Results.* The VISA-A score increased in both groups, from 49.57 ± 9.98 at baseline to 83.86 ± 8.59 at 14.5 ± 7.2 months after treatment in nondeformity group (*P* < 0.001) and from 48.70 ± 9.38 at baseline to 67.78 ± 11.35 at 15.3 ± 6.7 months after treatment in deformity group (*P* < 0.001). However, there was a greater improvement in VISA-A Score for the nondeformity group compared with deformity group (*P* = 0.005). For the 6-point Likert scale, there were decreases from 3.92 ± 0.80 at baseline to 1.57 ± 0.73 at the follow-up time point in nondeformity group (*P* < 0.001) and from 4.0 ± 0.76 at baseline to 2.37 ± 1.03 at the follow-up time point in deformity group (*P* < 0.001). There was no significant difference in improvement of the 6-point Likert scale between both groups (*P* = 0.062).* Conclusions.* ESWT resulted in greater clinical outcomes in patients without Haglund's deformity compared with patients with Haglund's deformity.

## 1. Introduction

Insertional Achilles tendinopathy (IAT) is among the most common posterior heel conditions while walking and running and is located at the insertion of the Achilles tendon onto the calcaneus, involving pain and swelling of the Achilles tendon itself, the formation of bone spurs, and calcifications at the insertion site [1]. Nonoperative management consists of rest, activity modification, anti-inflammatory medication, physical therapy, eccentric exercise, and corticosteroid injections [2]. Recent several studies have shown that extracorporeal shock wave therapy (ESWT) for the treatment of IAT has achieved good functional and clinical outcomes [3, 4]. Several fundamental studies have shown biological effects of ESWT for IAT. van der Worp et al. claimed that the nonexclusive theories about the mechanisms of ESWT involve pain relief, tissue regeneration, and destruction of calcifications [5]. Waugh et al. observed the increase of IL-6 and IL-8 which could promote fibroblast production of collagen and ECM components and demonstrated that the mechanical stimulus provided by ESWT might contribute to injured tendon tissue remodeling in tendinopathy [6]. Moreover, improved blood supply and early vascularity make use of ECM-degrading enzymes to promote the initial leukocyte infiltration and the subsequent metabolism of the fibers in the damaged tendon area [7]. The ESW-driven transitory increase in TGF-ß1 expression and persistent IGF-I expression could lead to some important changes including controlled inhibition of macrophages-induced ECM degradation and inflammation and an enhanced ECM and collagen type I synthesis [8]. The other therapeutic effects of ESWT consist of tendon cells proliferation and endogenous lubricin production by fibroblasts and tenocytes resulting from growth factors stimulation [9]. Therefore, the ESWT ultimately effectively bring about promotion of cell metabolism, and the latter may accelerate healing process in the pathological Achilles tendon tissue [10].

IAT may be associated with a Haglund's deformity, which is defined as a complex of symptoms involving a superolateral calcaneal prominence, retrocalcaneal bursitis, and superficial adventitious Achilles tendon bursitis [11–13]. The study of Sundararajan and Wilde exhibited that Haglund's deformity was present in 25% of IAT patients [13].

To our knowledge, it remains unclear if IAT concomitant with Haglund's deformity could achieve great clinical efficacy or not when treated with ESWT. Therefore, the purpose of this study was to reveal clinical outcomes following ESWT between IAT patients with or without Haglund's deformity. We hypothesized that patients with Haglund's deformity will have inferior clinical outcomes compared with patients without Haglund's deformity.

## 2. Materials and Methods

### 2.1. Participants

**Figure 1:**
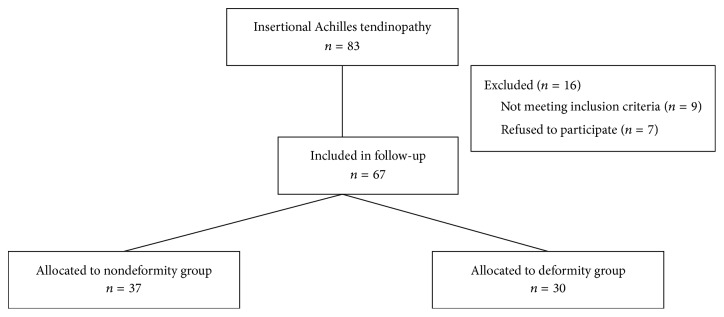
Flow chart of patients included in follow-up. Deformity: Haglund's deformity.

This study was approved by the Ethics Committee of Fudan University. Between September 2014 and May 2015, participants who received shock wave therapy for IAT were recruited retrospectively in this study. The diagnosis of IAT was confirmed by the following definition: pain and localized tenderness at the insertion region of the Achilles tendon and decreased activity levels secondary to Achilles tendon pain [3, 14–16]. All patients underwent preoperative lateral radiograph of ankle to identify Haglund's deformity. The diagnosis of Haglund's deformity was confirmed by the following definition: remarkable osseous prominence at the region of the posterosuperior part of the calcaneus and Fowler-Philip angle of >75° on lateral plain radiographs [3, 11]. According to the presence of Haglund's deformity, all patients were retrospectively classified into two groups: deformity group if patients have concomitant Haglund's deformity and nondeformity group if patients have no Haglund's deformity (Figure 1).

All patients included in this study failed to respond to nonsurgical treatment for at least six months. Nonoperative treatment included activity modification (*n* = 65), physiotherapy (*n* = 35), nonsteroidal anti-inflammatory drugs (*n* = 62), and the use of orthotics (*n* = 27). Steroid injections were not used because of the risk of Achilles tendon rupture and, in some cases, unwillingness to try. Exclusion criteria for this research included prior Achilles tendon rupture, previous surgery of the ankle or the Achilles tendon on the involved side, ankle arthritis, radiculopathy, or systemic neurological conditions, congenital or acquired deformities of the knee and ankle, peripheral neuropathy, lumbar radiculopathy, and inability to comply with the recommended treatment regimen.

### 2.2. Shock Wave Therapy

The shock wave therapy was performed with the patient in the prone position and was administered once a week, for 5 sessions. All patients completed all 5 sessions. And no patients received more sessions. A radial shock wave device (EMS Swiss Dolor-Clast, Munich, Germany) was used. Radial shock wave is created ballistically with the pressurized air source accelerating a bullet to strike a metal applicator. The kinetic energy produced is transformed into radially expanding shock waves from the application site into the tissue to be treated [3]. At each treatment session, 2000 pulses with an energy flux density of 0.12 mJ/mm^2^ and a rate of 8 pulses per second were applied. The applicator of hand-piece was located on the maximal tenderness point and was properly placed and adjusted according to patients' feedback during treatment if necessary [17]. No local anesthetic was applied.

### 2.3. Clinical Evaluation

**Figure 2:**
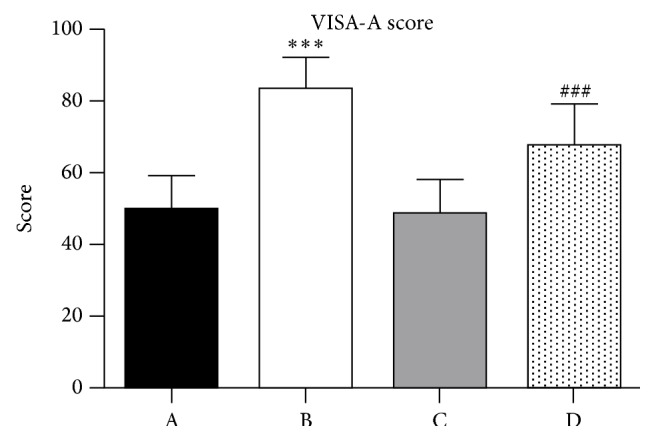
Results of VISA-A score for both groups. A: at baseline in nondeformity group. B: at follow-up in nondeformity group. C: at baseline in deformity group. D: at follow-up in deformity group. ^*∗∗∗*&###^ *P* < 0.001.

**Figure 3:**
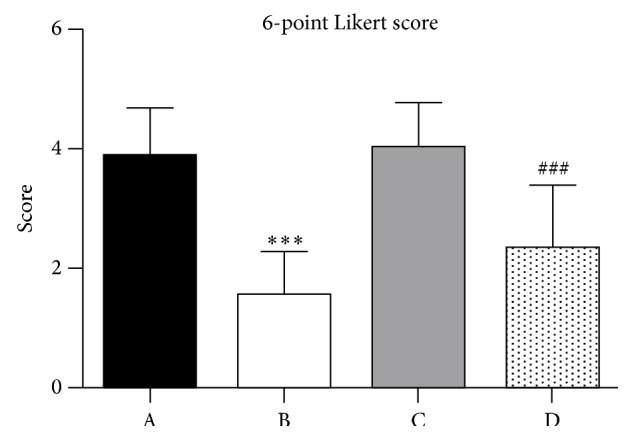
Results of 6-point Likert score for both groups. A: at baseline in nondeformity group. B: at follow-up in nondeformity group. C: at baseline in deformity group. D: at follow-up in deformity group. ^*∗∗∗*&###^ *P* < 0.001.

Patients, researchers evaluating the clinical outcome, and treating physicians were blinded to the presence of deformity or not. Clinical functional evaluation included VISA-A score (see Figure 2) and 6-point Likert scale (see Figure 3) collected before treatment and at the follow-up time point. As a self-administered questionnaire, the VISA-A score (Appendix) is used to evaluate the severity of Achilles tendinopathy. It has previously been shown to be valid, reliable, and clinically relevant [18]. The content of VISA-A questionnaire is as follows: pain (questions (1)–(3)), function (questions (4)–(6)), and activity (questions (7) and (8)). VISA-A scores have a range of 0 to 100. The 6-point Likert scale is interpreted as success if patients rate themselves 1 or 2 and as failure if patients rate themselves 3, 4, 5, or 6 [19].

### 2.4. Statistical Analysis

Data analysis is performed using Stata 10.0 software (StataCorp, College Station, TX, USA) and all data are expressed as mean and standard deviation (SD) for description.

The improvement between pretreatment and at the follow-up time point was calculated by paired *t*-test (see Figure 3). The difference between groups was compared by *t*-test. Statistical significance was set at *P* < 0.05. The odds ratio (OR) was calculated to figure out the influence of deformity on treatment failure. The 95% confidence interval (CI) was also calculated.

## 3. Results

### 3.1. Patient Demographics

At the follow-up time point, a total of 67 patients were available for follow-up, 30 patients in deformity group and 37 in nondeformity group. Participants' demographic data are shown in Table 1. The two groups did not differ significantly in age, body mass index, sex, therapeutic side, and follow-up time. All patients underwent extracorporeal shock wave therapy (ESWT).

### 3.2. Clinical Outcomes

At the follow-up time point, the functional outcomes with regard to VISA-A score and 6-point Likert scale achieved significant improvements in both groups. However, there was a greater improvement in VISA-A score for the nondeformity group compared with deformity group (*P* = 0.005) (Tables 2 and 3). According to the 6-point Likert scale grading system, there were 34 graded as success and 3 graded as failure in the nondeformity group, and there were 23 graded as success and 7 graded as failure in the deformity group. There were no serious complications including infection and Achilles tendon rupture, except transient reddening of the skin in all patients.

The OR was 3.45 with a 95% CI of [0.81,14.74], indicating that patients with deformity had a 3.45 times higher risk to experience treatment failure compared with those who are without. However, the difference was not significant (*P* = 0.09).

## 4. Discussion

The current study validated that ESWT for IAT concomitant with or without Haglund's deformity exhibited improved clinical outcomes. However, the VISA-A scores in patients with Haglund's deformity were inferior to those in patients without Haglund's deformity. The results of this study suggest that Haglund's deformity may worsen therapeutic effect of the ESWT for IAT.

**Figure 4:**
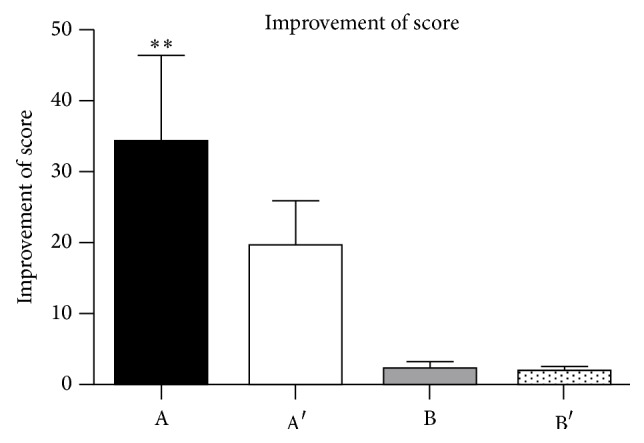
Improvement of clinical scores between nondeformity and deformity group. A: improvement of VISA-A in nondeformity group. A′: improvement of VISA-A in deformity group. B: improvement of 6-point Likert score in nondeformity group. B′: improvement of 6-point Likert score in deformity group. ^*∗∗*^ *P* = 0.005.

Recent numerous studies have reported that satisfactory clinical results in the treatments of IAT could be achieved with the use of ESWT [3, 4]. Furia concluded that shock wave therapy could obtain satisfactory clinical outcome in the treatment of the chronic insertional Achilles tendinopathy. Follow-up was performed at 1, 3, and 12 months after treatment, and the mean visual analog score for the nonoperative therapy and ESWT groups were 8.2 and 4.2 (*P* < 0.001), 7.2 and 2.9 (*P* < 0.001), and 7.0 and 2.8 (*P* < 0.001), respectively. Twelve months after treatment, more patients in the ESWT group (83% of ESWT group patients) have successful Roles and Maudsley scores compared to those in the control group [4]. In addition, in a randomized, controlled study, Rompe et al. showed shock wave therapy could provide more favorable results compared to eccentric loading for chronic IAT. At 4 months after treatment, both groups have improvement in the mean VISA-A score, increasing from 53 to 63 points in eccentric loading group and 53 to 80 points in shock wave therapy group. Moreover, this clinical result after shock wave therapy remained stable at the one-year follow-up evaluation [3]. In our present study, all the participants in both groups obtain pain relief and significant improvement in clinical outcomes (see Figure 4).

However, significant differences between both groups were observed in the current study, suggesting that Haglund's deformity in the posterior calcaneus has negative influence on clinical results of ESWT for IAT. The calcaneal posterosuperior prominence is called Haglund's deformity. Repetitive squeezing of the retrocalcaneal bursa at dorsal flexion of ankle, resulting from impingement between the posterosuperior prominence of the calcaneus and the anterior aspect of the Achilles tendon, can sometimes cause painful bursitis [20]. Sundararajan and Wilde found 25% frequency of Haglund's syndrome within the IAT population according to both the clinical examination and magnetic resonance imaging [13]. The previous study has reported that the presence of Haglund's deformity has influence on the clinical outcomes of other treatments including eccentric training, which was consistent with the results of our current study. Fahlström et al. found that only 32% of the patients with insertional Achilles tendon pain had good clinical results with usage of painful eccentric training beyond plantar grade. The unfavourable outcomes in patients with insertional Achilles tendon pain may be attributable to mechanical impingement between the prominent calcaneus and the tendon and bursa, when the ankle was in the dorsiflexed position [15]. Following the findings from Fahlström et al., Jonsson et al. made modification and used an eccentric training model without dorsiflexion in the ankle joint to avoid possible mechanical impingement, which resulted in 67% satisfied patients compared with the study of Fahlström et al. [21]. The presence of Haglund's deformity could explain the poorer clinical evaluation in deformity group compared with nondeformity group in the current study.

There are still some limitations in our study. The first is the small number of patients. Larger patient series are needed to verify whether the clinical outcomes could be undermined by Haglund's deformity. Secondly, a longer follow-up is necessary to determine whether ESWT for IAT with or without Haglund's deformity may persist in symptomatic relief. Lastly, a major limitation of the current study is the retrospective study-design, which makes it hard to perform a power analysis to ensure the statistical efficacy. Future trials with higher quality are required to reveal the influence of deformity on failure rates. The odds ratio (OR) was calculated to figure out the influence of deformity on treatment failure. We found that the OR was 3.45 with a 95% CI of [0.81,14.74], indicating that patients with deformity had a 3.45 times higher risk to experience treatment failure compared with those who are without. However, the difference was not significant (*P* = 0.09). Future trials with higher quality are required to reveal the influence of deformity on failure rates.

## 5. Conclusion

The clinical results of ESWT for IAT with and without Haglund's deformity showed significant improvement. However, IAT without Haglund's deformity had significantly greater VISA-A score compared with IAT with Haglund's deformity.

## Figures and Tables

**Table 1:** Participant demographic data of the study groups.

Variable	Nondeformity group (*n* = 37)	Deformity group (*n* = 30)	*P* value	95% CI
Age, mean ± SD, y	37.6 ± 9.2	35.8 ± 7.4	0.228	−2.17–5.17
Sex, mean ± SD, *n*			0.789	0.43–3.04
Male	21	18		
Female	16	12		
Body mass index, mean ± SD, kg/m^2^	23.7 ± 2.0	22.9 ± 2.2	0.591	−0.22–1.82
Therapeutic side, mean ± SD, *n*			0.818	0.42–2.89
left	15	13		
right	22	17		
Follow-up time, mean ± SD, months	14.5 ± 7.2	15.3 ± 6.7	0.705	−4.14–2.54

CI: confidence interval.

**Table 2:** Clinical outcome scores for both groups.

Outcome Score	Nondeformity group		*P* value	95% CI	Deformity group		*P* value	95% CI
	Baseline	Follow-up			Baseline	Follow-up		
VISA-A	49.57 ± 9.98	83.86 ± 8.59	<0.001	30.05–38.53	48.70 ± 9.38	67.78 ± 11.35	<0.001	13.81–24.35
6-point Likert	3.92 ± 0.80	1.57 ± 0.73	<0.001	2.00–2.70	4.0 ± 0.76	2.37 ± 1.03	<0.001	1.17–2.09

CI: confidence interval.

**Table 3:** Comparison of differences in improvement of clinical outcome scores between nondeformity and deformity group.

Improvement of clinical outcome scores				
	Nondeformity group	Deformity group	*P* value	95% CI
VISA-A	34.30 ± 11.96	19.08 ± 7.08	0.005	10.61–19.83
6-point Likert	2.29 ± 0.90	2.00 ± 0.64	0.062	−0.08–0.66

CI: confidence interval.

## Appendix

### The VISA-A Questionnaire: An Index of the Severity of Achilles Tendinopathy

In this questionnaire, the term pain refers specifically to pain in the Achilles tendon region

(1) For how many minutes do you have stiffness in the Achilles region on first getting up?

(2) Once you are warmed up for the day, do you have pain when stretching the Achilles tendon fully over the edge of a step? (keeping knee straight)

(3) After walking on flat ground for 30 minutes, do you have pain within the next 2 hours? (If unable to walk on flat ground for 30 minutes because of pain, score 0 for this question).

(4) Do you have pain walking downstairs with a normal gait cycle?

(5) Do you have pain during or immediately after doing 10 (single leg) heel raises from a flat surface?

(6) How many single leg hops can you do without pain?

(7) Are you currently undertaking sport or other physical activity?

(8) Please complete* EITHER (A), (B) or (C)* in this question.

(i) If you have* no pain while undertaking Achilles tendon loading sports* please complete* Q8a only*.

(ii) If you have* pain while undertaking Achilles tendon loading sports but it does not stop you from completing the activity*, please complete* Q8b only*.

(iii) If you have* pain that stops you from completing Achilles tendon loading sports*, please complete* Q8c only*.

(A) If you have* no pain* while undertaking* Achilles tendon loading sports*, for how long can you train/practise?

*OR*

(B) If you have some pain while undertaking* Achilles tendon loading sport*, but it does not stop you from completing your training/practice for how long can you train/practise?

*OR*

(C) If you have* pain that stops* you from completing your training/practice in* Achilles tendon loading sport*, for how long can you train/practise?
